# Extending bicluster analysis to annotate unclassified ORFs and predict novel functional modules using expression data

**DOI:** 10.1186/1471-2164-9-S2-S20

**Published:** 2008-09-16

**Authors:** Kenneth Bryan, Pádraig Cunningham

**Affiliations:** 1 Complex and Adaptive Systems Laboratory (CASL), University College Dublin, Belfield, Dublin 4, Ireland

## Abstract

**Background:**

Microarrays have the capacity to measure the expressions of thousands of genes in parallel over many experimental samples. The unsupervised classification technique of bicluster analysis has been employed previously to uncover gene expression correlations over subsets of samples with the aim of providing a more accurate model of the natural gene functional classes. This approach also has the potential to aid functional annotation of unclassified open reading frames (ORFs). Until now this aspect of biclustering has been under-explored. In this work we illustrate how bicluster analysis may be extended into a 'semi-supervised' ORF annotation approach referred to as BALBOA.

**Results:**

The efficacy of the BALBOA ORF classification technique is first assessed via cross validation and compared to a multi-class *k*-Nearest Neighbour (kNN) benchmark across three independent gene expression datasets. BALBOA is then used to assign putative functional annotations to unclassified yeast ORFs. These predictions are evaluated using existing experimental and protein sequence information. Lastly, we employ a related semi-supervised method to predict the presence of novel functional modules within yeast.

**Conclusion:**

In this paper we demonstrate how unsupervised classification methods, such as bicluster analysis, may be extended using of available annotations to form semi-supervised approaches within the gene expression analysis domain. We show that such methods have the potential to improve upon supervised approaches and shed new light on the functions of unclassified ORFs and their co-regulation.

## Background

Gene expression microarrays enable the expressions of thousands of genes to be measured in parallel over many experimental samples (growth conditions, time points, cell types etc.). The results from microarray experiments are generally presented in the form of an expression data matrix, where rows represent genes and columns represent samples (or vice versa depending on the experimental objective). Analysis of such gene expression data has shown that functionally related genes may have correlated expression profiles [1]. Sample profiles too, such as cell or disease types, often exhibit characteristic expression profiles [2]. From a data modelling perspective, a sample or gene profile may be thought of as a 'data object' with the gene or sample name representing the object's descriptor variable or *label *and the corresponding expression values representing the object's predictor variables or *features*. This raises the prospect of characterising and classifying genes or samples based on their expression profiles. In the case of experimental samples, such analysis is most often performed in relation to cell types e.g. the molecular characterisation of clinically similar cancer subtypes [2-4]. In this paper, however, we will focus on the functional classification of unannotated genes via their corresponding expression levels. Hereafter unannotated gene profiles will be referred to as 'open reading frame' (ORFs), rather than genes, as a functional protein product has yet to be verified.

Several modes of analysis may be applied to gene expression data depending on objectives of the study in question. Statistical methods such as *differential analysis *of gene expression over samples may be used to identify genes that show significantly different expression across sample classes. This can lead to the *ab initio *elucidation of gene function as well as the identification of key 'marker' genes whose expression are tightly correlated with sample classes [5]. Should sample or gene class labels be available, *supervised *machine learning methods may be applied to 'learn' the characteristic expression patterns of a class. Techniques such as *k*-nearest neighbour (kNN) and support vector machines (SVMs) have been applied successfully to classify both unlabelled genes and samples [6-8].

When class labels are unavailable, or perhaps debatable, *unsupervised *methods may be applied to attempt to model the class structure by analysing inter-object similarities with reference to features alone. *Cluster analysis *has been the most prevalent unsupervised method within the domain of expression data analysis and has been applied to model both sample and gene classes [1,9,10]. This technique typically separates the data into *k *disjoint groups of objects that have high similarity within groups and low similarity between groups. Expression similarity is best computed via a correlation based distance measure, such as Pearson's Correlation, rather than an absolute measure such as Euclidean distance, as such functionally related genes may be expressed at different absolute levels. In gene expression data analysis, genes exhibiting similar expression patterns may be co-regulated to perform a common function *in vivo*. Cluster analysis of genes therefore attempts to model the gene *functional modules *that exist within the expression data.

Conventional cluster analysis of genes computes expression similarity across the full set of sample features. However, as datasets increase in size it becomes increasingly unlikely, due to noise and measurement error that even functionally related genes will retain expression similarity over all experimental samples. Furthermore, some experimental samples may simply be irrelevant with regard to stimulating co-regulation within a gene functional module. As a result, measuring gene expression similarity exclusively over all samples has the potential to miss significant 'local' signals that may only be apparent over subsets of experimental samples.

To address this drawback, the 'two-way' clustering technique of *bicluster analysis *was proposed [11]. In this domain, biclustering involves grouping genes whose expression may correlate over a subset of experimental samples only. Apart from guarding against the prospect of missing significant local signals within the data, biclustering allows data objects to belong to more than one grouping, or to none at all. This aspect is also beneficial as a gene may belong to two or more functional modules expressed over different subsets of experimental conditions.

The question of how to best evaluate the class model retrieved by bicluster analysis is an important consideration. In some cases biclustering methods have been evaluated in terms of internal data-derived criteria alone [12-14]. Such evaluations however lack validation within the domain context. When applying unsupervised methods to gene expression data analysis any complete evaluation must include an assessment of the biological meaning of proposed classes. Generally, in expression data there are at least some gene labels available. As a result, bicluster analysis may be partially assessed in terms of 'functional enrichment' [15]. Functional enrichment is akin to the supervised machine learning term of *precision*, except of course we must decide upon our 'correct' label. The functional enrichment, *E*, of a bicluster may be given by *E *= *M/N*, where *M *is number of genes from the predominant functional category and *N *is the total number of genes in the bicluster. This simple metric may be extended to account for the distribution of the dominant class in the population by calculation of a *p*-value from the hypergeometric distribution (or in large populations its binomial approximation). However, in some cases, i.e. when the bicluster is large or it captures part of a relatively small class, a bicluster may have a seemingly significant *p*-value but a low, and uninformative, functional enrichment. As a result bicluster quality should be assessed using both measures.

Until now, the principal goal of bicluster analysis has been to reveal the natural underlying class structure (gene functional modules) within the expression data. Once this higher level class structure is modelled however, one may employ it to infer added functions for known genes or, more interestingly, assign functional labels to unclassified ORFs. The success of this latter objective clearly depends on the accuracy of the former class modelling approach. The utilization of the class model derived by bicluster analysis to aid functional annotation of unclassified ORFs has been under-explored. One possible method of assigning putative functions to unclassified ORFs is by examining biclusters in which these ORFs are present. If these biclusters have a high enrichment of genes from one functional category we might surmise that these unannotated ORFs may also belong to this category. This *guilt by association *method for ORF classification is discussed in the Methods section. In this paper we extend bicluster analysis to shift the focus onto unclassified ORF annotation rather than performing it as a secondary objective to class analysis. Referred to as BALBOA (**B**icluster **A**na**L**ysis **B**ased **O**rf **A**nnotation), this approach is a more systematic and directed method of predicting functional labels for unclassified ORFs using expression data. BALBOA's strength lies in the fact that it combines both the information on class structure, retrieved by bicluster analysis, and the available gene label information. In machine learning such approaches are often referred to as being 'semi-supervised' in nature.

BALBOA begins by partitioning the gene expression dataset into annotated genes and unannotated ORF subsets. Bicluster analysis is then performed on the annotated gene set alone. The resulting fully labelled biclusters are then assessed and used as 'classifiers' to label similarly expressed ORFs in the unannotated set. As BALBOA is based on bicluster analysis we briefly review this topic within this gene expression analysis domain in the Methods section. We then describe the BALBOA ORF classification strategy. An advantage of the BALBOA approach is that it may be evaluated via cross validation. In the Results section we compare BALBOA to the standard classification approach of *k*-nearest neighbour (kNN). The specific multi-class kNN implementations used are also described in this section. In this evaluation we use three independent gene expression datasets derived from *Saccharomyces cerevisiae *or budding yeast. In the second part of the Results section we then attempt to functionally classify yeast's unclassified ORFs using BALBOA. In the last part of the Results section we also attempt to model novel functional modules present within the unclassified ORF set.

Importantly, an advantage of using three independently generated gene expression datasets is that they represent different 'views' of the same transcriptome. Therefore any putative predictions concerning the functions of unclassified ORFs may be cross-referenced across datasets. This has the potential to significantly increase the support for both single annotations and predicted functional modules. We also attempt to support our putative annotations via external experimental and protein sequence information from the Saccharomyces Genome Database (SGD). We first give a brief review of bicluster analysis in the gene expression analysis domain.

## Methods

### Bicluster analysis of gene expression data

In the context of gene expression data, conventional cluster analysis involves computing similarity over all experimental samples, i.e. the full set of features. This approach may not be the most suitable for analyzing high dimensional gene expression datasets. Gene expression data may contain a significant amount of noise from sample variation and errors within the experimental measurement process itself. Furthermore, even the expressions of functionally related genes may not necessarily correlate over the full set of experimental samples. For example, co-regulation within a gene functional module may only be stimulated at certain stages in the cell cycle or environmental conditions. Cluster analysis, therefore, has the potential to miss significant subspace similarities within the data that may contribute to the modelling of the natural set of functional classes. To address this problem, the concept of biclustering was introduced to gene expression data analysis by Cheng and Church [11].

In a gene expression data matrix of genes and samples, a bicluster is defined as a subset of genes that show similar expression over a subset of experimental samples. In examining different sample subsets one can also see that different behaviours of the same gene can be captured. This aspect of biclustering enables it to model overlapping gene functional classes i.e. where genes have more than one function.

Cheng and Church proposed a bicluster scoring metric called the *mean squared residue (H) *to evaluate the correlation of the rows and columns within selected sub-matrices in the expression data matrix. This is given by:

(1) $$H(I,J)=\frac{1}{\left|I\right|\left|J\right|}\sum_{i\in I,j\in J}{\left(a_{ij}-a_{Ij}-a_{iJ}+a_{IJ}\right)}^{2}$$

where *a*_*ij *_is the entry at position *ij *in the sub-matrix (*I*, *J*), *a*_*iJ *_is the mean of the *i*th row, *a*_*Ij *_is the mean of the *j*th column and *a*_*IJ *_mean of the whole sub-matrix. A sub-matrix with a mean squared residue score below a chosen threshold (*δ*) is termed a *δ*-bicluster. The number of sub-matrices, *S*, (possible *δ*-biclusters) in an *M *× *N *matrix is:

(2) *S*_*MN *_= (2^*N *^- 1)(2^*M *^- 1)

In such an exponential search space an exhaustive search through all possible sub-matrix solutions is NP-hard [16] and therefore impracticable in large gene expression data matrices.

**Figure 1:**
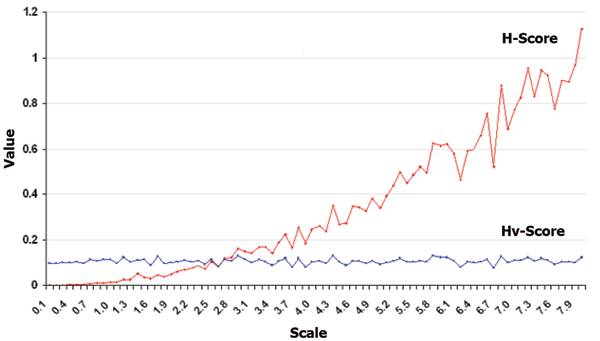
**Change in H-score and Hv-score with increasing bicluster scale**. Figure 1 illustrates how the H-score and improved Hv-score change as the scale of the bicluster being measured changes. Biclusters of different scales, but with the same relative row correlation, receive very different H-scores but approximately the same Hv-score. Biclusters were generated as in [28].

To tackle this issue Cheng and Church formulated the bicluster analysis problem as a 'greedy' search heuristic. The central iteration of this approach is a *node deletion *step that utilizes the means squared residue as the objective function. This seminal study spawned numerous directly related biclustering approaches [12,14,17] as well as conceptually similar strategies that incorporated alternative methods and metrics [18,19]. However, the mean squared residue was subsequently shown to contain some biases that directed searches towards less interesting biclusters containing genes with low expression variance [20], see Figure 1. Recently, we proposed BUBBLE (**B**ottom-**U**p **B**iclustering **B**y **L**ocality **E**xpansion) bicluster analysis strategy [21]. BUBBLE demonstrated improved results over the competitive graph theoretic approach, SAMBA, proposed by Tanay *et al. *(2002) over several gene expression datasets. BUBBLE begins by locating small, highly correlated regions within the gene expression data matrix via a *simulated annealing *based search method. These are referred to as *bicluster seeds*. Interestingly BUBBLE does not aim to find the global optimum only 'regional optima'. Furthermore, this method does not require selection on an arbitrary *δ *threshold. Importantly, BUBBLE utilizes a new metric, the *Hv*-score, which is unencumbered by the bias affecting the mean squared residue. This is defined by:

(3) $$Hv(I,J)=\frac{\sum_{i\in I,j\in J}{\left(a_{ij}-a_{Ij}-a_{iJ}+a_{IJ}\right)}^{2}}{\sum_{i\in I,j\in J}{\left(a_{ij}-a_{iJ}\right)}^{2}}$$

where *a*_*ij *_is the entry at position *ij *in the sub-matrix (*I*, *J*), *a*_*iJ *_is the mean of the *i*th row, *a*_*Ij *_is the mean of the *j*th column and *a*_*IJ *_is the mean of the sub-matrix. The *Hv*-score includes the row variance of the bicluster in the denominator which compensates for the bias inherent in the mean squared residue (equation 1).

In BUBBLE, once the bicluster seeds have been located they are then expanded in a deterministic manner by adding the most similar gene profiles to produce a full bicluster. The 'stopping' criterion for this expansion is based on the largest disimprovement in the *Hv*-Score of the growing bicluster.

BALBOA's bicluster analysis step is based upon the BUBBLE algorithm with some important extensions, discussed in the next section, designed to improve the subsequent ORF functional classification process. In the next section we shall examine the 'co-occurrence' method of ORF functional classification that has been thus far employed with both cluster and bicluster analysis of gene expression data.

### Unclassified ORF annotation via unsupervised class modelling

#### ORF annotation by co-occurrence analysis: 'guilt by association'

It is evident from previous cluster and bicluster analysis studies of gene expression data that functionally related genes tend to group together [1,22]. Extending this logic would suggest that similar functions be attributed to any unclassified ORFs also present. The support for this functional inference increases with the ratio of annotated to unclassified ORFs within the grouping and the functional enrichment of the annotated genes present. This co-occurrence analysis has also been referred to as the *guilt by association *approach [23].

Wu *et al. *applied several gene clustering techniques to expression data [15]. Using the above premise, they inferred putative functions for unannotated ORFs and labelled the clusters using the MIPS functional database (see Results section). To assess the precision of this strategy they iteratively re-labelled each annotated gene in turn as 'unclassified' and re-assessed all the cluster enrichments (and resultant ORF classifications). They achieved an accuracy of up to 80% with a recall of 40% in the case of the Protein Synthesis functional category. Although Wu *et al. *did some filtering on the basis of *p*-values they did not take into account the functional enrichment of the clusters, only whether the gene was present or not. Also, a significant number of the clusters contained many unannotated genes and therefore added limited information to the model. Lastly, as this annotation approach is built upon conventional clustering only, it does not utilize input from the gene relationships that only occur over significant subsets of experimental samples.

In another study, biclusters generated by the SAMBA biclustering algorithm were subsequently analyzed to produce a so called 'naive functional annotations' of unclassified ORFs [19]. Again *p*-value filtering was carried out but in this case the evaluation involved hiding 30% of the known annotations and then assessing the precision. Here the authors used the 10 Gene Ontology (GO) categories (rather than the more specific 17 MIPS categories). It was also noted that genes could be assigned more than one annotation as a result of this process. However, as *p*-values were used to select significant biclusters (*p *> 10^-4^) and no weight was given to functional enrichment of the biclusters, we can see that many annotations, some poorly supported, may be assigned to an ORF. It is also difficult to gauge the success of this approach as the authors only provide the *specificity *(percentage of true negatives correct) and the *selectivity *(recall) of their annotations. Both of the above classification approaches use a 'co-occurrence' strategy to putatively label unannotated ORFs. An alternative strategy might be to borrow from the 'labelled training set' approach used in supervised learning.

#### The BALBOA strategy for unclassified ORF annotation

In the BALBOA strategy for annotation of unclassified ORFs the gene expression dataset is first partitioned into labelled and unlabelled subsets, i.e. annotated genes and unannotated ORFs respectively. Gene labels are provided by the MIPS database, see Results section. Bicluster analysis is then applied to model the classes in the annotated subset. This produces fully labelled biclusters that can be used as 'classifiers' to direct labelling of the unclassified ORFs in the unannotated subset. This type of approach, in which both knowledge of labels and object similarities are combined to drive classification, may be referred to as 'semi-supervised' as the classification is partially directed by the labels that are available. This approach has several advantages over the 'co-occurrence' ORF annotation method described in the last section. Firstly, the biclusters are fully labelled, containing no unclassified ORFs, and therefore all will be more informative. The biclusters generated in this manner also tend to have higher functional enrichments than those generated over the total expression dataset. Furthermore, during evaluation the dataset may be partitioned into 'training' and 'testing' subsets enabling cross validation in accordance with standard practice (where training and testing sets remain separated) to be applied.

In BALBOA, the bicluster analysis step employs several extensions to the previous BUBBLE approach. Firstly, the diversity within the final bicluster population is increased by masking bicluster seeds as they are discovered. This masking involves replacing the entries of the dataset that represent the bicluster seed with random values from the inter-quartile range of the dataset. Selection from this range plus the fact that masking is limited to the bicluster seed minimizes the *random interference *referred to in [12]. Secondly, the number of bicluster solutions generated by BALBOA is also increased. As in ensemble clustering it is advantageous to increase the size and diversity of the set of biclusters as this will enable better determination of the most 'stable' relationships [24]. In this case however this stability adds increased support to our subsequent ORF classification step.

**Figure 2:**
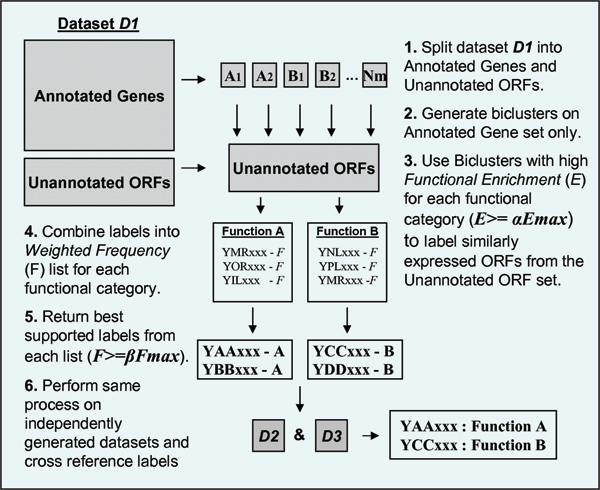
**Illustration of the steps in the BALBOA ORF classification algorithm**. This figure shows the various step of the BALBOA ORF prediction algorithm. In step 1 the expression dataset is divided into its annotated genes and unannotated (unclassified) ORFs. In step 2 biclusters are generated in the annotated gene set only. In step 3 selected biclusters (where *E *≥ *E*_*max*_) are used to classify similarly expressed ORFs in the unclassified set. In step 4 ORFs are combined into weighted frequency list for each functional category. Each ORF label weight is derived from the functional enrichments of the classifying biclusters. In step 5 the top ORFs (where *F *≥ *F*_*max*_) are selected from this list. In step 6 ORFs consistently classified across independent datasets are returned.

The full BALBOA strategy is illustrated in Figure 2. In step 1 the gene expression dataset is partitioned into *annotated *(genes with known functional labels) and *unannotated *(unclassified ORFs) subsets. In step 2 biclusters are then generated in the unannotated subset only. In step 3 biclusters that have relatively high functional enrichments, *E*, i.e. with *E *≥ *αE*_*max*_, where *α *is user defined and *E*_*max *_is the maximum functional enrichment for a functional category, are used to assign labels to similarly expressed unclassified ORFs in the unannotated subset.

In practice unclassified ORF profiles are first standardized (divided by their standard deviation). They are then sorted according to their mean squared residue score relative to the bicluster classifier. This relative profile scoring was first used by Cheng and Church in their *node deletion *approach. In this case, the stopping point for the annotation process is determined retrospectively by the largest 'jump' in dissimilarity that occurs as the ordered ORFs are presented to the classifier.

The label assigned to an unclassified ORF by a bicluster classifier is also weighted based on the functional enrichment of the bicluster. Unclassified ORFs may receive labels from more than one bicluster classifier. Where assigned labels agree weights are additively combined. So for example, if unclassified ORF *YAAxxx *is classified by two annotated biclusters with functional enrichments 0.90 amd 0.75 for the *Transcription *functional category, *YAAxxx *is assigned the label *Transcription *with a weight of 1.65. In step 4 this weighted frequency, *F*, is used to rank unclassified ORFs within lists for each functional category.

Unclassified ORFs that have many supporting biclusters with high functional enrichments for functional category *A *should top the weighted the list for category. In step 5, to reduce the potential for returning false positives, the best supported labels at the top of each list are then selected. We define a selection threshold, *F *≥ *βF*_*max*_, where *F*_*max *_is the highest weighted frequency in the list. Like the bicluster filtering parameter (*a*) the frequency threshold parameter, *β*, is user defined. In the extreme case, where *α *and *β *are set to 1.00, only unclassified ORFs labelled by the most enriched biclusters (*E*_*max*_) for each functional category and labelled most frequently (*F_max_*) will be assigned labels.

Since three yeast datasets are available in this study, a final cross-referencing step (step 6), where unclassified ORF labels are only assigned if they are consistent across multiple expression datasets, may also be performed.

As BALBOA involves separate 'training' and 'labelling' stages we may evaluate it via standard cross validation. Furthermore, although the set of unannotated ORFs are all officially designated as 'unclassified' by MIPS, for many there does exist some 'wet lab' experimental and/or protein sequence information. Although, evidently, this information is not sufficient to allow an official functional annotation it may still support the putative functional labels assigned by BALBOA.

## Evaluation

### Datasets

We use three yeast expression datasets in our evaluation. The 'Eisen' dataset contains 6,221 genes and 80 samples related to yeast cell-cycle, sporulation, and diauxic shift [1]. The 'Hughes' dataset contains 6,316 genes and 300 samples from an extensive functional analysis expression study [25]. The 'Gasch' dataset contains 6,129 genes and 150 samples from a yeast cell stress study [26]. Missing entries were randomly imputed from the inter-quartile range of each dataset. To reduce the impact of this imputation we removed all rows and columns containing extensive (≥ 25%) missing values.

ORF profiles were annotated via the MIPS (Munich Information Centre for Protein Sequences) database [27]. Approximately 1500 ORFs in yeast are assigned category 99 (Unclassified). These MIPS labels were used to evaluate the functional enrichment after our bicluster generation phase, allowing selection of good bicluster 'classifiers'.

Lastly, to avoid classification of non-coding ORFs those specified as 'dubious' (≈ 500) by the Saccharomyces Genome Database (SGD) were removed from each dataset. Datasets and algorithms are available on-line at .

### Multi-class k-NN implementations

Supervised approaches such as *k*-Nearest Neighbour (kNN) and Support Vector Machines (SVMs) have been used to classify unlabelled ORFs within expression data [6-8]. Recently, the 'local' kNN approach (that looks at the labels of the *k *most similar data object only) has been shown to out-perform the 'global' SVM approach (that searches for a class separating hyperplane) in the gene expression domain [23]. This is perhaps unsurprising as given the overlapping nature of gene functional classes a good class partition may be difficult to find.

In kNN an unlabelled 'query' object is compared with its *k *nearest labelled objects in feature space. This 'nearness' depends on its feature values and is defined by a suitable similarity metric. In our context the query consists of an unannotated ORF and its feature values are its expression levels. We use Pearson's correlation as the similarity metric. We use *k *= 10, which was previously used in this domain [23], and is also the number of genes present in the initial bicluster seed in BALBOA.

Also important in kNN is the voting process. Firstly, we use a majority voting scheme in which we assign the most prevalent label from our *k *nearest neighbours. We also implement a unanimous voting scheme in which we only assign a label to the query object if all *k *neighbours 'agree' or contain that label. This will reduce the *recall *but enable higher *precision *and reduce the potential for false positives. To cater for the possibility of a gene having multiple functional labels, we must implement these kNN in a multi-class manner. Therefore when two (or more) labels are equally prevalent among the *k *nearest neighbours we assign all labels to our query.

## Results

### Comparative cross validation with kNN

Before applying BALBOA to classify the unannotated yeast ORFs we must first determine its prediction accuracy, or *precision*, by cross validation. To do this we divide the annotated gene set into a *training set *and *test set*. We then train BALBOA on the training set and use the resultant bicluster classifiers to predict the functional labels of the test set. We perform two rounds of 4-fold cross validation. This 3:1 split reflects the natural annotated to unannotated ratio in yeast. In practice our *a *and *β *thresholds, described in the Methods section, are set as follows. Our set of best biclusters for each functional class are chosen by selecting biclusters with functional enrichment *E *≥ *αE*_*max*_, where *α *= 0.9 and *E*_*max *_is the maximal enrichment for a functional class. We combine the resultant labelled ORFs for each class into a weighted frequency list and select the best supported ORFs, such that *F *≥ *βF*_*max*_, where *β *= 0.9 and *F*_*max *_is the label with the highest weighted frequency. Although we could finely tune these parameters to achieve better results for each functional class these values allowed for best average predictions across all functional categories and all datasets. This fact may also reduce the possibility of over-fitting our training data. Cross validations for each yeast dataset are given in Table 1. Here we compare BALBOA's classification *precision *(P) and *recall *(R) with majority and unanimous voting multi-class kNN. In column one we see that majority kNN has a high recall but low precision and as a result its use as an ORF classifier is unfeasible. In column two we can see that unanimous voting markedly improves precision at the expense of recall. The final column shows that BALBOA out-performs unanimous kNN achieving a higher mean precision over all functional classes.

**Table 1:** Comparative cross validation of BALBOA with majority & unanimous voting kNN.

**MIPS Category**	**KNN**		**UkNN**		**BALBOA**	
**Eisen**	**P**	**R**	**P**	**R**	**P**	**R**

Metabolism (01)	0.46	0.43	0.50	0.00	**0.54**	0.01
Energy (02)	0.48	0.15	**0.63**	0.01	0.57	0.05
Cell Cycle (10)	0.48	0.41	0.86	0.03	**0.89**	0.02
Transcription (11)	0.47	0.36	0.13	0.00	**0.58**	0.03
Protein Synthesis (12)	0.51	0.52	0.97	0.24	**1.00**	0.08
Protein Fate (14)	0.38	0.26	**0.94**	0.01	0.91	0.02
Transp. Elements (38)	0.29	0.57	0.25	0.09	**0.50**	0.43
Cell Fate (43)	0.30	0.09	0.00	0.00	**0.58**	0.05

Mean	0.42	0.35	0.53	0.05	**0.70**	0.09
						
**Hughes**	**P**	**R**	**P**	**R**	**P**	**R**

Metabolism (01)	0.47	0.50	0.85	0.03	**0.85**	0.04
Energy (02)	0.59	0.16	**0.98**	0.03	0.76	0.08
Cell Cycle (10)	0.49	0.24	**0.85**	0.01	0.57	0.03
Transcription (11)	0.44	0.43	0.62	0.01	**0.81**	0.03
Protein Synthesis (12)	0.52	0.47	0.95	0.18	**1.00**	0.05
Protein Fate (14)	0.39	0.24	**0.50**	0.00	0.47	0.02
Prot. Bind. Func. (16)	0.29	0.14	0.00	0.00	**0.57**	0.01
Cell Transport (20)	0.36	0.20	**0.80**	0.02	0.50	0.03
Transp. Elements (38)	0.38	0.48	0.00	0.00	**0.77**	0.47
Biogen. Cell. Comp. (42)	0.32	0.15	0.41	0.01	**0.74**	0.04

Mean	0.43	0.31	0.60	0.03	**0.70**	0.09
						
**Gasch**	**P**	**R**	**P**	**R**	**P**	**R**

Metabolism (01)	0.46	0.49	**0.88**	0.01	0.83	0.03
Energy (02)	0.52	0.20	**0.94**	0.05	0.83	0.09
Cell Cycle (10)	0.46	0.29	**0.75**	0.00	0.58	0.03
Transcription (11)	0.45	0.37	0.64	0.00	**0.74**	0.06
Protein Synthesis (12)	0.50	0.55	0.95	0.31	**0.98**	0.17
Protein Fate (14)	0.40	0.29	**1.00**	0.03	0.93	0.01
Cell Transport (20)	0.36	0.20	0.25	0.00	**0.66**	0.01
Transp. Elements (38)	**0.79**	0.52	0.00	0.15	0.75	0.50
Biogen. Cell. Comp. (42)	0.33	0.12	0.38	0.00	**0.60**	0.03

Mean	0.48	0.34	0.64	0.06	**0.77**	0.10

The highest precisons for each MIPS category in each dataset evaluated are shown in bold. BALBOA achvieves the highest mean precision over all MIPS categories in each dataset. P = Pecision, R = Recall.

As opposed to kNN, BALBOA allows for the capturing of signals that occur over subsets of the experimental features in the dataset. In the *Transcription (11) *functional category in particular this facet seems to allow for a greater classification precision to be achieved across all three datasets. In other categories such as *Protein Synthesis (12) *UkNN is more competitive. This is not surprising as it is well known that genes in this fundamental functional category are strongly co-expressed over most conditions.

### Functional annotation of unclassified ORFs

Now that we have determined BALBOA's classification precision for each functional class we can apply it to classify unannotated yeast ORFs. Heretofore our evaluation has focused on the top level MIPS categories (*01, 02 etc.*). However in some cases it may be possible to assign a more specific and informative functional label. A prime example is that of *Protein Synthesis (12)*. Expression correlations of genes from this category are most often due to the highly co-regulated sub-category of *ribosomal proteins (12.01.01)*. In this study we have also observed that this ribosomal co-regulation is also evident in the ribosomal RNA (rRNA) processing genes. This sub-category, specifically *rRNA processing (11.04.01)*, often dominates the biclusters that are functionally enriched for *Transcription (11)*. These sub-category labels are provided where available and allow for a more specific putative annotation.

Of the 954 unclassified ORFs, BALBOA made putative functional predictions for 135 from the Eisen expression data, 113 from the Gasch data and 119 from the Hughes data. These span 13 of the 17 MIPS functional categories. Interestingly, despite the different experimental conditions investigated in the three independent expression studies, 21 annotations, spanning 7 MIPS functional categories, were consistent across 2 or more datasets. We will now focus on these consistent annotations, presented in Table 2. We can see from this table that some of BALBOA's putative functional annotations seem to be well supported given the external experimental and sequence information. One case which stands out in particular is that of YCR072C. This ORF was unclassified by MIPS when we labelled the data. However this ORF has subsequently been labelled by MIPS as being involved in transcription of ribosomal RNA (rRNA) and assigned category *rRNA processing (11.04.01)*. This functional label agrees with our predicted function. In fact six of the seven ORFs assigned to the *Transcription (11) *functional category seem to have good supporting external evidence. This includes YDL167C and YJR003 both of which are supported by previous computational evidence defined as Reviewed Computational Evidence (RCA) by the SGD. Another notable annotation is that of YIL060W. This ORF is putatively assigned to *Transposable Elements (38) *functional category and is the only ORF given the same annotation across all three datasets. Interestingly, the translation of this ORF seems to have a significant similarity (*p*-value = 2.2e^ -21^) to retrotransposons TyA Gag and TyB Pol genes. This would seem to corroborate our putative annotation. The two unclassified ORFs labelled as *ribosomal proteins (12.01.01) *also have additional labels supported over two datasets. In the case of YLR196W both predicted functions appear to be supported given that this ORF is already thought to be involved in rRNA processing.

**Table 2:** BALBOA annotation of unclassified ORFs that are consistent over two or more datasets.

**ORF**	**Predicted Function**	**Experimental Evidence (SGD)**	**Protein Sequence**
YJR154W	Metabolism: Amino Acid (01.01)	Putative protein, unknown function. GFP-fusion protein localizes to cytoplasm.	Similarity (*p *= 2.8e^-5^) to cytosolic L-asparaginase (YDR321W).
YDL072C (YET3)	Energy: Respiration (02.13)	Null mutant has decreased level of secreted invertase (enables respiration of sucrose).	Human BAP31 homolog.
YGR149W	Energy: Respiration (02.13)	Putative protein, unknown function	Predicted integral membrane protein.
YCR072C (RSA4)	Transcription: rRNA (11.04.01)	Recently verified by MIPS – ribosomal biogenesis.	
YDL167C (NRP1)	Transcription: rRNA (11.04.01)	Role in ribosome biogenesis and assembly (RCA).	
YMR259C	Transcription: rRNA (11.04.01)/*Transport Routes (20.09)*	Putative protein, unknown function; GFP-fusion protein localizes to the cytoplasm.	
YNL022C	Transcription: rRNA (11.04.01)	Putative protein of unknown function. GFP-fusion protein localizes to a single spot in the nucleus.	Similarity (*p *= 1.2e^-18^) to YNL061W involved in rRNA processing.
YDR361C (BCP1)	Transcription (11)/*Ribosomal Proteins (12.01.01)*	Associated with RPL23a & PL23b (Ribosomal sub-units) in Affinity Capture Expts.	
YJL122W (ALB 1)	Transcription (11)/*Ribosomal Proteins (12.01.01)*	Shuttling pre-60S factor; involved in the biogenesis of ribosomal large subunit.	
YJR003C	Transcription: rRNA (11.04.01)/*Transport Routes (20.09)*	Putative protein. Detected in purified mitochondria. Role in ribosome biogenesis and assembly (RCA).	
YLR196W (PWP1)	Ribosomal Proteins (12.01.01)/Transcription (11)	Protein with WD-40 repeats involved in rRNA processing.	
YER049W (TPA1)	Ribosomal Proteins (12.01.01)/Cellular Transport (20)	Interacts with Sup45p (eRF1) and Sup35p (eRF3) and Pab1p; role in translation termination efficiency.	
YDR282C	Transported Compounds(20.01)/*Tansposable Elements (38)*	Putative protein of unknown function.	Similarity (*p *= 2.2e^-21^) to YDL001W required for sporulation.
YGR266W	Transport Routes (20.09)	Protein of unknown function. Localizes to mitochondrial outer membrane and plasma membrane	Predicted to have single trans-membrane domain.
YIL039W	Transport Routes (20.09)	GFP-fusion localizes to the ER. Deletion confers sensitivity to GSAO (angiogenesis inhibitor drug).	
YOR175C	Transport Routes (20.09)	Protein of unknown function. Co-purification with Ribosomes.	Member of MBOAT putative membrane bound O-acyltransferases.
YPL105C	Transport Routes (20.09)/*Transcription (11)*	Protein of unknown function. Co-purification with both Ribosomes & mitochondria.	
YIL060W	Tansposable Elements (38)	Putative protein of unknown function. Mutant accumulates less glycogen than does wild type.	Similarity (*p *= 4.2e^-06^) to TyA & TyB Retrotransposons.
YJR030C	Tansposable Elements (38)	Putative protein of unknown function. Expression repressed in carbon limited cultures	Similar to YJL181w (cell cyle regulator) & MBP-1 binding site (cell cycle).
YIL157C	Biogenesis of Cellular Components: Mitochondrion (42.16)	Detected in Co-purified mitochondria. Null mutant is defective in cytochrome oxidase.	
YML030W	Biogenesis of Cellular Components: Mitochondrion (42.16)	Putative protein of unknown function; GFP-fusion protein localizes to mitochondria.	

Unclassified ORFs consistently annotated over two or more datasets, additional labels in italics are from one dataset only. GFP = Green Fluorescent Protein; Rca = Reviewed Computational Analysis.

Of BALBOA's other putative annotations, the ORF assigned to *amino acid metabolism (01.01)*, YJR154W, has been localized to the cytosol by green fluorescent protein (GFP) fusion localization experiments. Furthermore, the translation of YJR154W has some small similarity (*p*-value = 2.8e^-5^) to cytosolic L-asparginase. Two ORFs were putatively assigned to *respiration (02.13) *functional category. Of these the YDL072C null mutant shows decreased levels of secreted *invertase*. Invertase is an enzyme involved in converting sucrose into glucose and fructose, a required step if sucrose to be used as an energy source. Of the ORFs assigned to the *transport routes (20.09) *category the best supported seems to be YIL039W. The fact that deletion of this ORF confers sensitivity to the drug GSAO might suggest a possible involvement in the export of this compound. Lastly, the two ORFs putatively annotated as *Biogenesis of Cellular Components: mitochondrion (42.16) *are actually both localized to the mitochondrion, YIL157C by co-purification and YML030W by GFP-fusion. The YIL157C null mutant also shows some defect in cytochrome oxidase, a mitochondrial enzyme involved in generating the proton gradient needed for ATP synthesis.

Intuitively, annotations that correspond across two independent datasets should have more support than those from a single dataset only. One method of quantifying this cross dataset support is the *union of probability *rule for two corresponding independent events, given by *P*_1 _⋃ *P*_2 _= *P*_1 _+ (1 - *P*_1_)(*P*_2_). As a result, even the least supported annotations, i.e. those of *Cellular Transport (20) *with cross validation precision 0.50 and 0.66, become more significant when consistently supported over two datasets, increasing to 0.83. With better supported classes, such as *Transcription (11)*, such cross referencing achieves precision values close to 1.

The *α *and *β *values we use in BALBOA may be somewhat stringent, especially if we include the dataset cross referencing step. However, looking at the final list, we see that most functional annotations seem to be supported by external evidence. One may consider this final cross referencing step a more valid selection procedure than the *β *selection threshold, as it is derived from the data. Interestingly, we find that upon reducing *β *to 0.5, 56 ORF annotations then agree across two or more datasets. This includes 12 that agree across all three. However, that being said, our chosen *α *and *β *values are perhaps more conducive to minimizing false positives and also proving the efficacy of the BALBOA classification approach via external corroboration. In this section we applied BALBOA to annotate individual unclassified ORFs. In the next section we look at a related semi-supervised strategy of predicting novel functional modules within the unclassified ORFs.

### Discovery of novel functional modules

In the previous section functional labelling of individual unclassified ORFs was performed. The best supported annotations, that agreed across two or more datasets, were presented along with supporting experimental and protein sequence evidence. In this section we aim to analyse the similarly expressed groups within the set of unclassified ORFs in an attempt model unclassified functional modules or at least unclassified parts of known functional modules.

**Figure 3:**
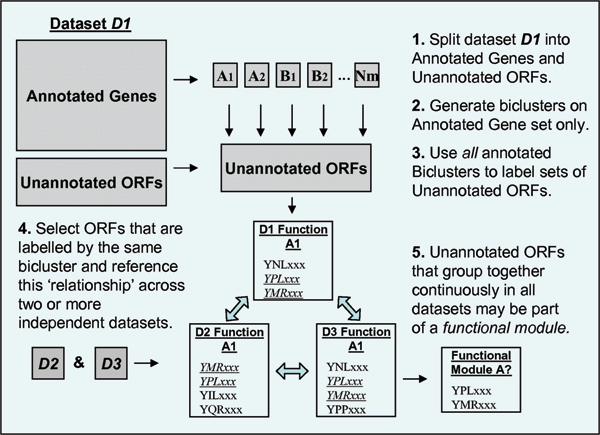
**Illustration of the steps in the semi-supervised functional module discovery process**. This figure shows the various step of the functional module discovery algorithm. This algorithm is related to the BALBOA algorithm and begins in the same manner with the dataset splitting in step 1 and the bicluster analysis of the annotated genes only in step 2. In step 3 however all biclusters are selected for analysis. In step 4 dataset cross validation is carried out to establish groups of ORFs that are consistently grouped together by biclusters over the three datasets. In step 5 these consistently grouped unclassified ORFs are returned as predicted functional modules, where the function may be inferred by the enrichment and significance of the dominant functional class in the classifying biclusters.

As in the previous section we first employ an initial dataset partitioning in which the expression data is split into its annotated genes and unannotated ORFs. Again this allows the class specific gene expression patterns (labelled biclusters) to be used as classifiers. The full process is illustrated in Figure 3. In this process each annotated bicluster generally labels several similarly expressed ORFs from the unannotated set. Unlike BALBOA we now employ dataset cross-referencing at this stage to discover which ORF groups are maintained across multiple independent data views. Groups of unclassifed ORFs, consistently associated together across independent expression datasets may represent novel functional modules. The nature of these predicted functional modules may then be gauged by reference to the functional enrichment of the annotated biclusters. The largest unclassified ORF groupings retrieved in this manner are presented in Table 3.

**Table 3:** Predicted functional modules of unclassified ORFs.

**ORF**	**Physical/Genetic Associations (SGD)**	**Functional Evidence**
**Ribosomal RNA procesing (11.04.01)**		

YBR271W	Localizes to the cytoplasm	S-adenosylmethionine-dependent methyltransferase.
YCR016W	YGL120C (PRP43) RNA helicase/maturation of rRNA, YPR135W (CTF4) Chromatin-associated protein	
YDL063C	YPL131W (RPL5-Protein of (60S) ribosomal subunit)	GO ribosome biogenesis & assembly (RCA)
YDL167C (NRP1)		GO ribosome biogenesis & assembly (RCA)
YDR361C (BCP1)	Protein component of the large (60S)	GO ribosomal large subunit export from nucleus (IMP); Export of Mss4p lipid kinase
YGR187C (HGH1)	YDR188W (CCT6-Chaperonin Containing TCP-1)	GO ribosome biogenesis & assembly (RCA)
YIL064W		GO ribosome biogenesis & assembly (RCA). S-adenosylmethionine-dependent methyltransferase
YIL096C	YLR009W (RLP24) 60S ribosomal subunit biogenesis	GO ribosome biogenesis & assembly (RCA)
YIL110W		Putative S-adenosylmethionine-dependent methyltransferase
YIL127C	Localizes to the nucleolus	GO ribosome biogenesis & assembly (RCA)
YJR003C	Detected in purified mitochondria	GO ribosome biogenesis & assembly (RCA)
YLR051C (FCF2)		Essential nucleolar protein, 35S rRNA processing
YLR196W (PWP)	YPL131W (RPL5-Protein of (60S) ribosomal subunit), YDR188W (CCT6-Chaperonin Containing TCP-1)	GO rRNA processing (IMP, ISS)
YLR287C	YPR135W (CTF4) Chromatin-associated protein	
YOL022C		Null mutant accumulates 20S pre-rRNA
YOR021C (TSR4)		GO ribosome biogenesis & assembly (RCA)
YOR154W (SLP1)		SUN like protein
YOR252W (TMA16)	YLR009W (RLP24) 60S ribosomal subunit biogenesis, YGL120C (PRP43) RNA helicase/maturation of rRNA	
YPL183C		Negative regulation of transposition, RNA-mediated (IMP)

**DNA Topology (10.01.02)**		
YBL112C		Contained within telomere TEL02L, TEL02L-YP
YEL076C		Contained within telomere TEL05L, TEL05L-YP, YEL076C-A
YER189W		Contained within telomere TEL05R, TEL05R-YP
YHR219W		Putative protein of unknown function with similarity to helicases; Contained within telomere TEL08R, TEL08R-YP

**Mitochondrial (42.16) & Ribosomal Proteins (12.01.01)**		
YDR493W		Null mutant displays decreased frequency of mitochondrial genome loss
YKL137W		Mutation results in growth defect on a non-fermentable (respiratory) carbon source
YLR204W	Mitochondrial inner membrane protein	
YLR218C		Growth defects on a non-fermentable carbon source
YML030W	Localizes to mitochondria	Null mutant is viable & displays decreased frequency of mitochondrial genome loss
YMR157C	detected in purified mitochondria	Displays increased frequency of mitochondrial genome loss

Predicted functional modules of unclassified ORFS. Genetic/Physical interaction and functional evidence supporting predicted functional modules. GO = Gene Ontology; RCA = Reviewed Computational Analysis.

**Figure 4:**
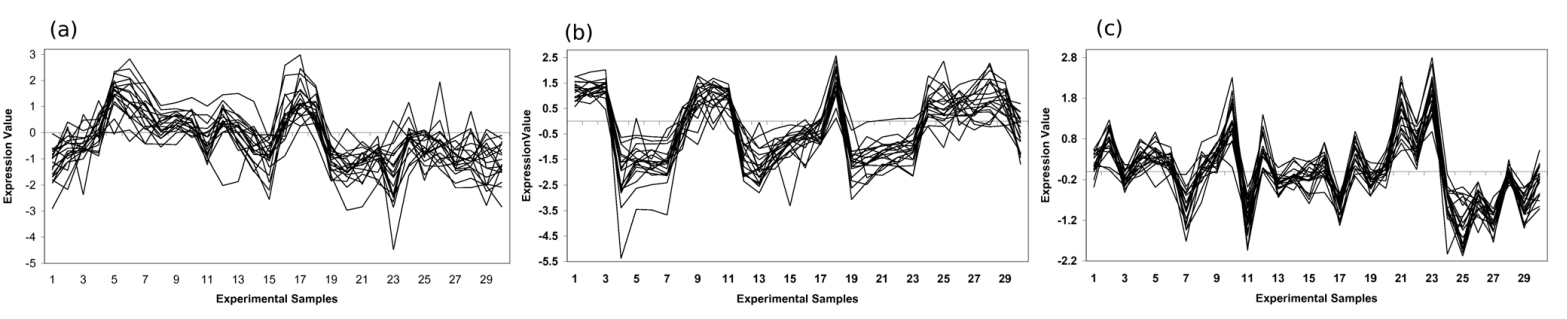
**Predicted functional module supported by biclusters enriched with Transcription: Ribosomal RNA processing (11.04.01)**. This figure shows the largest group of unclassified ORFS that were consistently classified together by biclusters significantly enriched for *Transcription: Ribosomal RNA processing (11.04.01) *in three independent datasets. The 19 unclassified ORFs in this predicted functional module correlate tightly over a subset of 30 sample features in the (a) Eisen, (b) Gasch and (c) Hughes expression datasets.

By far the largest group of unclassified ORFs associated together in the Eisen, Gasch and Hughes expression datasets was a set of 19 ORFs: YBR271W, YCR016W, YDL063C, YDL167C, YDR361C, YGR187C, YIL064W, YIL096C, YIL110W, YIL127C, YJR003C, YLR051C, YLR196W, YLR287C, YOL022C, YOR021C, YOR154W, YOR252W, YPL183C. As we can see from Figure 4 this set is highly correlated across all three datasets. The nature of this possible functional module may be inferred by examining the functional enrichment of the annotated biclusters used to predict this set in each dataset. In all three datasets the predicting annotated bicluster was significantly functionally enriched for genes from the *Ribosomal RNA processing (11.04.01) *functional category with *p*-values of 2.61 × 10^-11^, 1.53 × 10^-14 ^and 1.24 × 10^-8 ^in the Eisen, Gasch and Hughes datasets respectively. Eisen and Gasch biclusters were also enriched to a lesser extent in *Ribosome biogenesis (12.01) *genes, 2.66 × 10^-6 ^and 2.08 × 10^-7 ^respectively. Interestingly, YDL167C, YDR361C, YJR003C and YLR196W were individually predicted to be involved in these processes by BALBOA in the last section. Further physical/genetic association and functional evidence from the SGD is provided in Table 3.

**Figure 5:**
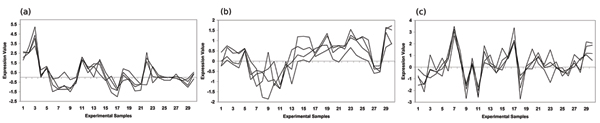
**Predicted functional module supported by biclusters enriched with DNA Topology (10.01.02)**. This figure shows a group of unclassified ORFS that were consistently classified together by biclusters significantly enriched for the *DNA Topology (10.01.02) *in three independent datasets. The 4 unclassified ORFs in this predicted functional module correlate tightly over a subset of 30 sample features in the (a) Eisen, (b) Gasch and (c) Hughes expression datasets.

Another set of unclassified ORFs consistently grouped together across the three expression datasets is YBL112C, YEL076C, YER189W, YHR219W These are predicted by annotated biclusters highly enriched for the *DNA Topology (10.01.02) *functional category with *p*-values of 7.26 × 10^-29^, 6.58 × 10^-14 ^and 2.89 × 10^-7 ^in the Eisen, Gasch and Hughes expression datasets respectively. The correlated expression of this group of unclassified ORFs, across all three datasets, can be seen in Figure 5. In Table 3 we see that these ORFs are located in the telomeric regions and YHR219W has some similarity to helicases.

**Figure 6:**
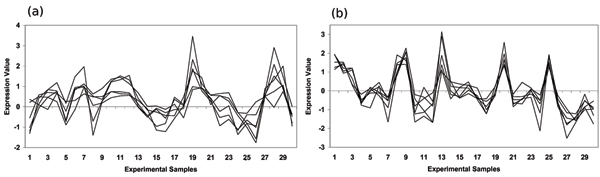
**Predicted functional module supported by biclusters enriched with Mitochondrial (42.16) & Ribosomal Proteins (12.01.01)**. This figure shows a group of unclassified ORFS that were consistently classified together by biclusters significantly enriched for the *Mitochondrial (42.16) *& Ribosomal Proteins (12.01.01) in two independent datasets. The 6 unclassified ORFs in this predicted functional module correlate tightly over a subset of 30 sample features in (a) Gasch and (b) Hughes expression datasets.

Another set of unclassified ORFs, consistently predicted by annotated biclusters across the Hughes and Gasch datasets is YDR493W, YKL137W, YLR204W, YLR218C, YML030W, YMR157C. These annotated biclusters were highly enriched in *Mitochondrial (42.16) *and *Ribosomal Proteins (12.01.01) *with significances of 2.48 × 10^-53 ^& 5.88 × 10^-40 ^and 1.23 × 10^-36 ^& 4.23 × 10^-34 ^for these functional categories in the Hughes and Gasch datasets respectively. The correlated expression of this group of unannotated ORFs can be seen in Figure 6 and additional supporting interaction and functional evidence is shown in Table 3.

## Discussion

BALBOA represents a novel, systematic approach for ORF classification using expression data. BALBOA's novelty lies both in the use of biclustering for training and classification and also in the subsequent 'ensemble-like' prediction strategy in which information provided by multiple biclusters is combined. Although such ensemble techniques are conceptually simple, resulting predictions tend to be more robust. In the first part of the Results section, cross validation showed that, on average, BALBOA achieves improved results over multi-class implementations of majority kNN and the more competitive unanimous kNN. Unlike kNN, BALBOA caters for 'local' correlations over feature subsets. This seems to allow for markedly improved classification precision for certain functional categories such as *Transcription (11)*. This thus demonstrates the necessity for classification approaches in this domain to provide for the prospect of sub-space similarities. kNN is still a powerful technique when features are relevant and it may prove interesting in future work to perform kNN with those features selected by bicluster analysis. In the second part of the Results section, we saw that BALBOA's ORF predictions were well supported by external functional evidence from the Saccharomyces Genome Database (SGD). Strong support, in particular, was provided by the recent official MIPS annotation of YCR072C. Although these annotations are putative they may still aid in the design of improved 'wet lab' experiments that may in turn lead to official annotation.

In the last part of the Results section, we examined groups of unclassified ORFs consistently predicted together across two or more independent datasets with the aim of modelling functional modules present within the set of yeast unclassified ORFs. The most prominent signal was the large correlated set of 19 ORFs labelled as *rRNA processing (11.04.01)*. Cross dataset analysis and additional supporting functional evidence supports this grouping and predicted function and this may be a good candidate set for further 'wet-lab' analysis.

It is common to combine data from individual expression studies into one large dataset prior to analysis. However, we have shown that maintaining multiple perspectives or data 'views' allows dataset cross referencing and potentially increases support for findings. Also, sample sets from different sources may reveal different ORF functions. Maintaining multiple perspectives may also prove useful in standard bicluster and cluster analyses. Lastly, this work demonstrates the benefit of applying newly developed data mining techniques to re-examine expression data after initial studies. In this paper we demonstrate the potential new insights, in this case into unclassified ORF function and functional modules, that this re-assessment may provide.

## Competing interests

The authors declare that they have no competing interests.

## Authors' contributions

KB and PC developed the concepts which motivated the BALBOA algorithm. KB developed the algorithms and evaluation and drafted the manuscript. PC advised on the methodology used in the evaluation section of the paper. All authors read and approved the final manuscript.
